# Retinal Oxygen Metabolism and Haemodynamics in Patients With Multiple Sclerosis and History of Optic Neuritis

**DOI:** 10.3389/fnins.2021.761654

**Published:** 2021-10-12

**Authors:** Martin Kallab, Nikolaus Hommer, Andreas Schlatter, Gabriel Bsteh, Patrick Altmann, Alina Popa-Cherecheanu, Martin Pfister, René M. Werkmeister, Doreen Schmidl, Leopold Schmetterer, Gerhard Garhöfer

**Affiliations:** ^1^Department of Clinical Pharmacology, Medical University of Vienna, Vienna, Austria; ^2^Vienna Institute for Research in Ocular Surgery (VIROS), Karl Landsteiner Institute, Hanusch Hospital, Vienna, Austria; ^3^Department of Neurology, Medical University of Vienna, Vienna, Austria; ^4^Carol Davila University of Medicine and Pharmacy, Bucharest, Romania; ^5^Department of Ophthalmology, University Emergency Hospital, Bucharest, Romania; ^6^Center for Medical Physics and Biomedical Engineering, Medical University of Vienna, Vienna, Austria; ^7^Institute of Applied Physics, Vienna University of Technology, Vienna, Austria; ^8^Singapore Eye Research Institute, Singapore, Singapore; ^9^Nanyang Technological University, Singapore, Singapore; ^10^Ophthalmology and Visual Sciences Academic Clinical Program, Duke-NUS Medical School, Singapore, Singapore; ^11^SERI-NTU Advanced Ocular Engineering (STANCE), Singapore, Singapore; ^12^Institute of Molecular and Clinical Ophthalmology, Basel, Switzerland

**Keywords:** multiple sclerosis, retinal blood flow, retinal oxygen saturation, microcirculation, optical coherence tomography angiography

## Abstract

Vascular changes and alterations of oxygen metabolism are suggested to be implicated in multiple sclerosis (MS) pathogenesis and progression. Recently developed in vivo retinal fundus imaging technologies provide now an opportunity to non-invasively assess metabolic changes in the neural retina. This study was performed to assess retinal oxygen metabolism, peripapillary capillary density (CD), large vessel density (LVD), retinal nerve fiber layer thickness (RNFLT) and ganglion cell inner plexiform layer thickness (GCIPLT) in patients with diagnosed relapsing multiple sclerosis (RMS) and history of unilateral optic neuritis (ON). 16 RMS patients and 18 healthy controls (HC) were included in this study. Retinal oxygen extraction was modeled using O_2_ saturations and Doppler optical coherence tomography (DOCT) derived retinal blood flow (RBF) data. CD and LVD were assessed using optical coherence tomography (OCT) angiography. RNFLT and GCIPLT were measured using structural OCT. Measurements were performed in eyes with (MS+ON) and without (MS-ON) history for ON in RMS patients and in one eye in HC. Total oxygen extraction was lowest in MS+ON (1.8 ± 0.2 μl O_2_/min), higher in MS-ON (2.1 ± 0.5 μl O_2_/min, *p* = 0.019 vs. MS+ON) and highest in HC eyes (2.3 ± 0.6 μl O_2_/min, *p* = 0.002 vs. MS, ANOVA *p* = 0.031). RBF was lower in MS+ON (33.2 ± 6.0 μl/min) compared to MS-ON (38.3 ± 4.6 μl/min, *p* = 0.005 vs. MS+ON) and HC eyes (37.2 ± 4.7 μl/min, *p* = 0.014 vs. MS+ON, ANOVA *p* = 0.010). CD, LVD, RNFLT and GCIPL were significantly lower in MS+ON eyes. The present data suggest that structural alterations in the retina of RMS patients are accompanied by changes in oxygen metabolism, which are more pronounced in MS+ON than in MS-ON eyes. Whether these alterations promote MS onset and progression or occur as consequence of disease warrants further investigation.

**Clinical Trial Registration:** ClinicalTrials.gov registry, NCT03401879.

## Introduction

Multiple sclerosis (MS) is a demyelinating disease of the central nervous system with a global median prevalence of approximately 33 people per 100,000 and one of the major reasons for permanent disability in young adults (Reich et al., 2018). Although there is general agreement that MS is an immune-mediated process, there is compelling evidence that vascular factors and metabolic alterations such as mitochondrial dysfunction (Dutta et al., 2006; Mahad et al., 2008) or hypoxia (Davies et al., 2013; Yang and Dunn, 2015; Johnson et al., 2016) play an essential role in the pathogenesis and progression of the disease. As such, it has been shown that local oxygen supply insufficiency leads to hypoxic damage, resulting in neuroinflammation and demyelination of nerve fibers with the known clinical consequences, while in turn neuroinflammation per se can also trigger hypoxia (Yang and Dunn, 2019; Halder and Milner, 2021). Thus, more knowledge on the oxygen metabolism may help to get a better understanding of the pathophysiological mechanisms involved in the disease progression and develop new therapeutic strategies.

Currently, studying microvascular and metabolic changes in MS is hampered by a paucity of available non-invasive methods to measure oxygen metabolism in the human brain. In this context, the anterior visual system – mainly the neural retina and the optic nerve – offers unique possibilities to non-invasively gain insight into the metabolic and microvascular processes in unprecedented resolution. Recent development in retinal imaging allows for the non-invasive determination of oxygen saturation in retinal vessels (Hammer et al., 2008) as well as for the quantitative assessment of perfusion (Werkmeister et al., 2008) and microvascular density (Tan et al., 2020). Further, as a part of the central nervous system, the retina is a highly metabolically active tissue, which is frequently affected by MS. More specifically, in 15–20% of patients diagnosed with MS, the symptom leading to clinical investigation is optic neuritis (ON) (Confavreux et al., 2000). Further, as much as 70% of patients with MS are affected by ON at some time during the disease (Toosy et al., 2014).

The current study takes advantage of recently developed imaging techniques to assess retinal oxygen metabolism, retinal perfusion and capillary density in patients with diagnosed relapsing MS (RMS) and history of unilateral ON and to compare these parameters with a healthy control group. Retinal oxygen extraction is measured based on Doppler optical coherence tomography (DOCT) (Werkmeister et al., 2008) and non-invasive determination of oxygen saturation via reflectometry (Hammer et al., 2008). This approach has been validated and recently successfully used to investigate metabolic changes under pathological conditions such as diabetes (Fondi et al., 2017). Further, structural changes were assessed using optical coherence tomography (OCT) and OCT angiography (OCTA).

The aim of this observer-masked cross-sectional study was to investigate potential metabolic and vascular alterations of the retina in patients with MS.

## Materials and Methods

### Study Subjects

MS patients as well as age- and sex-matched healthy subjects were recruited between February 2018 and January 2021. The study was conducted in accordance with the Declaration of Helsinki and the Good Clinical Practice (GCP) guidelines of the International Council for Harmonisation of Technical Requirements for Pharmaceuticals for Human Use (ICH). Written informed consent was obtained from all participants before any study related procedures and the Ethics Committee of the Medical University of Vienna approved the study with all its procedures before initiation.

### In/Exclusion Criteria

Inclusion criteria for MS patients were age ≥18 years, diagnosis of relapsing multiple sclerosis (RMS) according to McDonald criteria (revision 2017), history of unilateral ON with unaffected contralateral eye, ON more than one year ago, adequate visual and auditory acuity to allow ocular blood flow measurements, stable doses of concomitant medications for at least 30 days prior inclusion if considered relevant by the investigator, normal ophthalmologic findings apart from MS- or ON-related alterations and ametropia < 6 Dpt.

Exclusion criteria for MS patients were presence or history of a severe medical condition other than MS as judged by the clinical investigator, history of neuromyelitis optica spectrum disorder (NMOSD), history of any inflammatory or infectious disease of the central nervous system other than MS, any other significant and clinically relevant neurological disease as judged by the investigator, untreated arterial hypertension and diabetes. Further, patients with ocular diseases or presence of any abnormality preventing reliable measurements in the study eyes as judged by the investigator, best-corrected visual acuity (BCVA) < 0.5 Snellen, pregnancy or planned pregnancy and alcoholism or substance abuse were excluded.

Inclusion criteria for healthy subjects were age over 18 years, normal findings in the medical history (or clinically irrelevant as judged by the investigator), normal ophthalmic findings and ametropia <6 Dpt. Exclusion criteria for healthy subjects were: history of any disease of the central nervous system, presence or history of any severe medical condition as judged by the investigator, untreated arterial hypertension, presence of any abnormalities preventing reliable measurements in the study eye as judged by the investigator, family history of MS, ON, or NMOSD, BCVA < 0.5 Snellen, pregnancy or planned pregnancy and alcoholism or substance abuse.

### Experimental Paradigm

Before inclusion into the study, a screening examination was performed to assess eligibility. It comprised the following procedures: detailed medical history including assessment of current MS-related symptoms (in MS patients) and concomitant medication, pregnancy testing in women with childbearing potential, measurement of systemic haemodynamics, BCVA, visual field (VF) testing using standard automated perimetry (SAP; Humphrey 30-2 SITA-Standard, Carl Zeiss Meditec Inc., Dublin, Ireland), measurement of axial eye length using an IOL-Master (Carl Zeiss Meditec Inc.), slit-lamp examination including dilated funduscopy and measurement of intraocular pressure (IOP) using applanation tonometry.

Upon confirmation of eligibility, patients and healthy subjects were included into the study. One drop of tropicamide was administered to the study eye(s) and a 20-min resting period was scheduled to obtain stable haemodynamic conditions. Then, an ONH-centered 50° fundus image was taken using a retinal vessel analyzer (RVA; Imedos Systems, Jena, Germany) to measure vessel diameters and O_2_ saturations, before retinal blood flow was assessed using a previously described custom built dual-beam DOCT system (Werkmeister et al., 2008; Doblhoff-Dier et al., 2014). Finally, a 3.5 diameter circumpapillary OCT ring scan, a 20°×20° macular volume scan and a 15°×15° peripapillary OCTA volume scan (384 B-Scans and 384 A-Scans/B-Scan) using the commercial Heidelberg Spectralis OCT(A) (Heidelberg Engineering, Heidelberg, Germany) were performed. In MS patients, both the eye with (MS+ON) and without (MS-ON) history for ON were measured, in healthy subjects one randomly chosen eye was measured.

### Methods

#### Noninvasive Measurement of Systemic Haemodynamics

Measurements of systemic haemodynamics were performed on the upper arm by an automated oscillometric device (Infinity Delta; Dräger, Vienna, Austria). This device recorded systolic, diastolic and mean arterial pressures (SBP, DBP, MAP), pulse rate (HR) and peripheral oxygen saturation using a fingertip pulse oximeter.

#### Intraocular Pressure

A slit-lamp mounted Goldmann applanation tonometer was used to assess IOP at the screening examination. One drop of oxybuprocainhydrochloride combined with sodium fluorescein was used for anesthesia of the cornea before each measurement.

#### Circumpapillary Optical Coherence Tomography, Macular Optical Coherence Tomography and Peripapillary Optical Coherence Tomography Angiography

Circumpapillary OCT scans were analyzed using the segmentation and analysis software of the Spectralis glaucoma module and global RNFL-thickness (RNFLT) in μm was extracted for every measurement.

Macular OCT scans were used to measure the ganglion cell layer and inner plexiform layer thickness (GCIPL) as previously described using the standard Spectralis software segmentation of GCL and IPL (Bsteh et al., 2021). In short, GCL and IPL thicknesses in the inner quadrants (3 mm) of the macula-centered ETDRS grid were averaged and GCIPL was calculated as the sum of the averaged GCL and IPL thicknesses.

Peripapillary OCTA scans were processed using standard segmentation and slab settings of the Spectralis OCTA module. Superficial vascular complex (SVC) slabs were exported to the Fiji distribution of ImageJ (Schindelin et al., 2012). Major retinal vessels were separated from the capillary bed using a Hessian-based large vessel detection algorithm (Sato et al., 1998) as frequently applied in the analysis of retinal OCTA scans (Tan et al., 2020). In short, a Hessian-based filter captures tubular structures of a certain caliber range and the output was used to create a binary vessel mask.

For capillary-specific analysis, this mask was applied to remove major retinal vessels before an optic disc-centered ring-shaped region of interest (ROI) with an inner diameter of 2.5 mm and an outer diameter of 4 mm was defined. The ring-shaped, capillary-specific ROI was binarized by mean values and capillary density (CD) was calculated as percentage of white pixels. For large vessel-specific analysis, the inverted vessel mask was applied to remove the capillaries before ROI definition, thresholding and density calculation was done as described for the capillary-specific analysis to determine the large vessel density (LVD).

#### Retinal Vessel Diameter and Oxygen Saturation

The RVA system allows for the evaluation of retinal vessel diameters and oxygen saturation (Hammer et al., 2008; Garhofer et al., 2010). For this purpose, a computer-coupled fundus camera is used.

Applying the VesselMap software to the ONH-centered fundus image as acquired by the RVA, all peripapillary arteries and veins branching from the central retinal artery (CRA) and central retinal vein (CRV) were selected and the CRA- and CRV-equivalent (CRAE and CRVE) and arterio-venous ratio (AVR) were calculated by the software as proposed previously (Hubbard et al., 1999).

Fundus images as taken using the RVA device were also used to estimate oxygen saturations of all retinal arteries (SaO_2,A_) and veins (SaO_2,V_) using a reflectometric approach (Hammer et al., 2008, 2009). In short, two images with different wavelengths are simultaneously taken (610 and 545 nm) and oxygen saturation is estimated based on the fact that hemoglobin exerts different light absorption characteristics depending on its level of oxygenation. While this effect is greatest at around 610 nm, the isosbestic wavelength for hemoglobin i.e., the wavelength at which deoxygenated and oxygenated hemoglobin show identical absorption characteristics is 548 nm. Using the differences of these two images, SaO_2,A_ or SaO_2,V_ are estimated in all retinal vessels.

#### Total Retinal Blood Flow Measurement

For TRBF calculation, measurements of the above-mentioned fundus-camera coupled DOCT device were used. As described previously, a peripapillary scanning pattern including horizontal and vertical scans was applied to ensure coverage of all retinal arteries and veins and blood flow was assessed in all vessels with a diameter of 40 μm or larger (Doblhoff-Dier et al., 2014). Each scan consisted of repetitive B-scans at the same position and lasted for several seconds to ensure averaging of parameters over a minimum of one full pulse cycle. The background and details of single vessel velocity, diameter and flow extraction have been extensively described in numerous previous publications (Werkmeister et al., 2012a,b, 2015; Doblhoff-Dier et al., 2014; Fondi et al., 2017; Bata et al., 2019). In reference to these papers, we confine ourselves to a short summary: two orthogonally polarized superluminescent diode beams separated by the known angle Δα are focused onto one retinal spot. Due to the two different angles α_1_ and α_2_(Δα = α_1_−α_2_) at which the two probe beams impinge onto the vessel of interest, the phase shifts Φ_1_ and Φ_2_ induced into the probing light reflected by moving particles (e.g., red blood cells) are different. This difference (ΔΦ(ΔΦ = Φ_1_−Φ_2_) between the two probe beams in combination with Δα, several device and physiological constants (λ = OCT light source central wavelength, τ = time interval between two recordings dependent on acquisition rate, n = group refractive index of blood) and the angle β (angle between the detection plane spanned by the two probe beams and the velocity vector) can be used to calculate absolute blood velocity using the following equation (Eq. 1):


(1) $$V_{a\,b\,s}=\Delta \,\Phi *\frac{\lambda }{4\,\pi *n*\tau *\Delta \,\alpha *\cos\beta }$$

Vessel diameter (*D*) is extracted from the DOCT scans using a caliper in the analysis software and absolute flood flow (*Q*_*abs*_) is calculated using Eq. 2


(2) $$Q=\frac{D^{2}}{4}*\pi *V_{a\,b\,s}$$

Summing up single vessel flow results for all arteries and veins gives arterial and venous TRBFs (TRBF_A_ and TRBF_V_). TRBF measurements have been recently evaluated for reproducibility and repeatability (Szegedi et al., 2020b). TRBFs presented in this study are the means of TRBF_A_ and TRBF_V._

#### Oxygen Content and Retinal Oxygen Extraction

The model to calculate the oxygen content and retinal oxygen extraction based on SaO_2,A_ and SaO_2,V_ was profoundly described and discussed previously by Werkmeister et al. (2015) and has since been applied in further studies in healthy subjects as well as in patients with ocular diseases (Fondi et al., 2017; Bata et al., 2019; Hommer et al., 2021). In short, SaO_2,A_ and SaO_2,V_ are corrected for the distance of their measurement point to the CRA or CRV merging point. By calculating the mean of the corrected SaO_2,A_ values (cSaO_2,A_), oxygen saturation in the central retinal artery (SaO_2,CRA_) can be directly obtained. For calculation of oxygen saturation in the central retinal vein (SaO_2,CRV_), an additional step taking the flow in each individual vessel as weighting factor into account is necessary as the venous blood in the CRV is a mixture of all merging retinal veins. In a next step, the oxygen content of the CRA (cO_2,CRA_) and CRV (cO_2,CRV_) are estimated considering the fact that not only hemoglobin bound oxygen needs to be considered but also the oxygen dissolved in plasma.

Finally retinal oxygen extraction (extO_2_) is calculated using Eq. 3, where cO_2,CRA_ and cO_2,CRV_ are the oxygen contents of CRA and CRV, respectively and Q is the TRBF as described above.


(3) $$e\,x\,t\,O_{2}=\left(c\,O_{2,C\,R\,A}-c\,O_{2,C\,R\,V}\right)*Q$$

### Statistical Analysis

Statistical analysis was performed using IBM SPSS Statistics (Version 27, IBM, Armonk, NY, United States). All values are presented as means ± standard deviations. Normal distribution was confirmed using the Kolmogorov-Smirnov test. Descriptive statistics are reported for all values obtained. A one-way ANOVA model was used to assess overall differences between the three groups (MS+ON, MS-ON and healthy eyes). For those that turned out to be significant in the ANOVA model for three groups, contrasts between two groups (MS+ON vs. MS-ON, MS+ON vs. healthy eyes and MS-ON vs. healthy eyes) were defined. Prior to calculating contrasts for planned comparison between groups, a Levene’s test to assess equality of variances was carried out. Plots for the figures were produced using GraphPad Prism 9.2.0 (GraphPad Software Inc., CA, United States). A *p*-value <0.05 was considered as the level of significance.

## Results

A total of 34 subjects were included in the present study, of which 16 had MS and 18 were healthy age- and sex-matched controls. As both eyes were measured in MS patients with history of unilateral ON a total of 16 MS+ON, 16 MS-ON and 18 healthy eyes were enrolled in this study. The demographics and baseline characteristics of the two study groups are shown in Table 1. There was no difference between groups in terms of age, sex or systemic haemodynamics.

**TABLE 1:** Baseline characteristics of the two study groups.

	**Healthy subjects**	**Patients with MS**		***p*-value**
Age (years)	41 ± 16	43 ± 13		0.698
Gender (m/f)	4/14	4/12		0.583
Years since diagnosis of MS (years)	N/A	9 ± 8		N/A
Time elapsed since ON (years)	N/A	8 ± 7		N/A
Systolic blood pressure (mmHg)	117 ± 14	123 ± 13		0.197
Diastolic blood pressure (mmHg)	72 ± 8	76 ± 8		0.128
Heart rate (bpm)	66 ± 9	69 ± 12		0.446
Mean arterial pressure (mmHg)	90 ± 12	96 ± 10		0.145
Intraocular pressure (mmHg)	13 ± 2	15 ± 3 (MS+ON)	15 ± 3 (MS-ON)	0.062
Best corrected visual acuity (Snellen decimal)	1.2 ± 0.5	1.2 ± 0.3 (MS+ON)	1.2 ± 0.2 (MS-ON)	0.937
Visual field mean deviation (dB)	−0.5 ± 1.2	−1.8 ± 3.2 (MS+ON)	−1.0 ± 2.9 (MS-ON)	0.333

*Values are presented as mean ± standard deviation.*

*ON, optic neuritis; MS, multiple sclerosis.*

*p-values are calculated by one-way ANOVA.*

Eleven (11) out of 16 MS patients were currently medicated with a disease-modifying therapy, of which five took glatiramer acetate, three dimethyl fumurate and one each fingolimod, natalizumab or interferon beta.

Seven (7) patients reported no current MS-related symptoms, 6 reported one and 3 reported more than one symptom. The most frequent symptoms were upper/lower limb paresthesia, gait disorder, fatigue and vertigo.

### Retinal Vessel Diameters

CRAE was significantly different between the three groups (*p* = 0.015), with CRAE being significantly lower in MS+ON eyes (182 ± 13 μm) and MS-ON eyes (182 ± 16 μm) compared to healthy eyes (196 ± 18 μm, *p* = 0.014 for MS+ON vs. healthy and *p* = 0.011 for MS-ON vs. healthy). In contrast, no significant difference between groups was found for CRVE (222 ± 15 μm in MS+ON eyes, 223 ± 13 μm in MS-ON eyes and 227 ± 14 μm in healthy eyes (*p* = 0.649 between groups). AVR was also significantly different between groups (*p* = 0.034). It was significantly reduced in eyes with MS+ON compared to healthy eyes (0.82 ± 0.06 vs. 0.87 ± 0.07, *p* = 0.047) as well as in MS-ON eyes compared to healthy eyes (0.81 ± 0.06 vs. 0.87 ± 0.07, *p* = 0.015).

### Oxygen Extraction and Total Retinal Blood Flow

**FIGURE 1:**
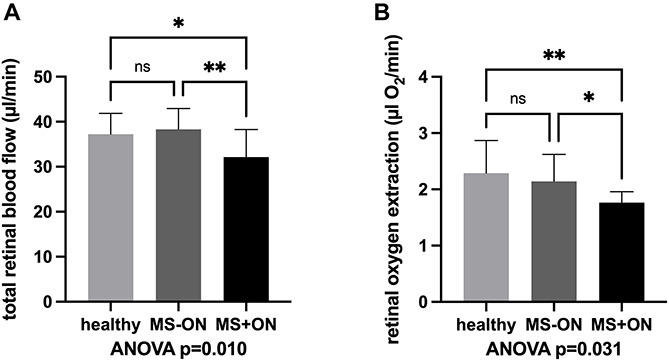
Total retinal blood flow **(A)** and retinal oxygen extraction **(B)** in eyes with history of acute optic neuritis (MS+ON), in contralateral eyes with no history of ON (MS-ON) and healthy control eyes are significantly different between the study groups upon ANOVA analysis. MS+ON eyes showed significantly reduced total retinal blood flow and oxygen extraction as compared to MS-ON and healthy eyes. Data are presented as means ± standard deviation. ^∗^*p* < 0.05, ^∗∗^*p* < 0.01, ns, not significant.

A statistically significant difference between the three groups was found for TRBF (*p* = 0.010, Figure 1A) and calculated oxygen extraction (*p* = 0.031, Figure 1B). Calculated oxygen extraction was lowest in MS+ON eyes (1.8 ± 0.2 μl O2/min), higher in MS-ON eyes (2.1 ± 0.5 μl O2/min; *p* = 0.019 for MS+ON vs. MS-ON, Figure 1B) and highest in healthy eyes (2.3 ± 0.6 μl O2/min; *p* = 0.002 for MS+ON vs. healthy, Figure 1B). TRBF was lower in MS+ON eyes (33.2 ± 6.0 μl/min) as compared to MS-ON eyes (38.3 ± 4.6 μl/min) and healthy eyes (37.2 ± 4.7 μl/min; *p* = 0.005 for MS+ON vs. MS-ON eyes, *p* = 0.014 for MS+ON vs. healthy eyes, *p* = 0.560 for MS-ON vs. healthy eyes, Figure 1A).

### Retinal Nerve Fiber Layer Thickness, Ganglion Cell Inner Plexiform Layer Thickness, Capillary Density and Large Vessel Density

RNFLT and GCIPL were significantly different between the three groups (*p* < 0.001 each). RNFLT was significantly lower in MS+ON eyes (80.7 ± 14.0 μm) compared to MS-ON eyes (96.8 ± 8.9 μm) and eyes of healthy controls (100.0 ± 9.5 μm; *p* < 0.001 between MS+ON and MS-ON or healthy eyes, *p* = 0.311 between MS-ON and healthy eyes). This was also similar for GCIPL which was significantly lower in MS+ON eyes (73.7 ± 14.6 μm) compared to MS-ON eyes (92.2 ± 9.4 μm, *p* < 0.001 vs. MS+ON eyes) or healthy eyes (93.8 ± 7.2 μm, *p* < 0.001 vs. MS+ON eyes; *p* = 0.665 vs. MS-ON eyes).

**FIGURE 2:**
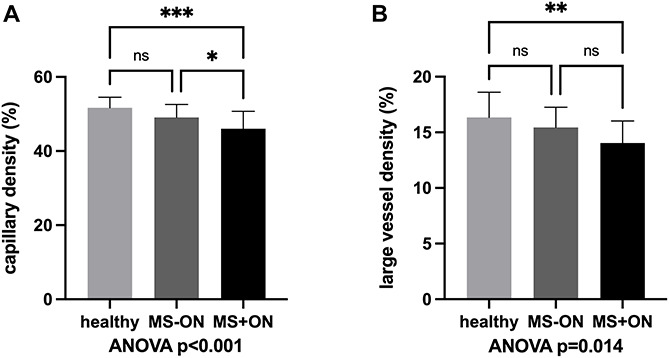
Capillary density **(A)** and large vessel density **(B)** in eyes with history of acute optic neuritis (MS+ON), in contralateral eyes with no history of ON (MS-ON) and healthy control eyes are significantly different between the study groups upon ANOVA analysis. MS+ON eyes showed a significantly reduced capillary density as compared to MS-ON and healthy eyes while large vessel density was significantly different between healthy and MS+ON eyes, only. Data are presented as means ± standard deviation. ^∗^*p* < 0.05, ^∗∗^*p* < 0.01, ^∗∗∗^*p* < 0.001, ns, not significant.

Peripapillary CD without large vessels as assessed by OCTA was significantly different between the three groups (*p* < 0.001). In particular, it was lower in MS+ON eyes (46.0 ± 4.7 %) compared to MS-ON eyes (49.1 ± 3.5 %, *p* = 0.037 vs. MS+ON eyes) and compared to healthy eyes (51.7 ± 2.9 %, *p* < 0.001 vs. MS+ON eyes). No statistically significant difference was found between MS-ON eyes and healthy eyes (*p* = 0.069, Figure 2A). LVD also showed a significant difference between the three groups (*p* = 0.014). It was significantly reduced in MS+ON eyes compared to healthy eyes (14.0 ± 2.0% vs. 16.3 ± 2.3%, *p* = 0.004), but no significant differences were found between MS+ON and MS-ON eyes (14.0 ± 2.0% vs. 15.4 ± 1.8%, *p* = 0.075) or MS-ON and healthy eyes (*p* = 0.236, Figure 2B).

No statistically significant correlations were found between changes in hemodynamic parameters and RNFL/GCIPL loss in neither MS+ON nor MS-ON eyes (data not shown).

## Discussion

To the best of our knowledge, the data from our study shows for the first time that retinal oxygen extraction is reduced in patients with MS and history of unilateral ON when compared to healthy subjects, suggesting an impaired oxygen metabolism in patients with MS and ON. Further, our results indicate that these impairments are more pronounced in the ON eye compared to the fellow eye without history of ON and paralleled by a decrease in retinal blood flow. Finally, our data confirms previous evidence for microvascular rarefication in patients with MS compared to healthy controls.

As the eye offers ideal opportunities to observe microvascular changes in-vivo, extensive research has been performed to investigate changes in the retinal neural tissue in patients with MS (Petzold et al., 2017; Britze and Frederiksen, 2018; Kleerekooper et al., 2020). As such, early post-mortem studies indicate that the anterior visual pathway is involved in 90% of patients with MS (Toussaint et al., 1983). More recently, OCT has been used as a non-invasive technique to assess potential neurodegenerative changes in different layers of the neural retina (Petzold et al., 2017). This was done in an effort to investigate whether this technique might provide potential novel biomarkers for neurodegeneration in patients with MS (Garcia-Martin et al., 2017; Britze and Frederiksen, 2018). Interestingly, the latter studies indicate that retinal thinning is present in patients with MS, independently of a history of ON and with the most pronounced differences in the peripapillary RNFL and macular ganglion cell layer and inner plexiform layer (Petzold et al., 2017). Although these anatomic changes in the retina of patients with MS are well described, there is only sparse knowledge on functional changes. Although recent reports indicate impaired perfusion in patients with MS (Wang et al., 2018; Liu et al., 2019), the question whether TRBF or oxygen metabolism is altered in patients with MS is not yet answered.

The present study provides in-vivo evidence that retinal oxygen metabolism is compromised in patients with MS. In particular, we found that retinal oxygen extraction was lowest in MS+ON eyes, higher in MS-ON eyes and highest in healthy eyes. The reason for this reduction of oxygen extraction is not entirely clear, but may be related to a reduced oxygen demand in particular of the inner retina caused by neurodegenerative changes of retinal neurons.

However, when discussing these findings, the complex oxygen supply of the retina needs to be considered. The retina is nourished by two distinct vascular beds: the retinal circulation, which supplies the inner retina including the ganglion cells and their associated axons and the choroidal circulation, which provides oxygen and other nutrients mainly to the photoreceptors of the outer retina. As the oxygen diffusion from the choroid to the inner retina is assumed to be negligible under physiological conditions, changes in the oxygen extraction of the retinal circulation can be mainly attributed to inner retinal oxygen consumption (Linsenmeier and Zhang, 2017). Hence, our findings may be at least partially explained by the reduced number of retinal neural cells and their axons, which are nourished via the retinal circulation. Along this line of thought, we have recently shown that oxygen extraction as measured by the model used in the current study correlates with the RNFLT as measured using structural OCT, and the total number of retinal ganglion cells in healthy subjects (Bata et al., 2019). This indicates that a loss of retinal neural cells may lead to a reduced oxygen demand of the tissue and consequently to a reduced oxygen extraction. This hypothesis is also supported by the results of the current study. Our results show a reduced GCIPL and RNFLT as well as reduced capillary density and arteriolar narrowing in patients with MS, indicative for a structural loss in this groups of patients. Further, these findings are also in keeping with previous results reporting that eyes of patients with MS and ON show a reduced RNFLT and GCIPL (Walter et al., 2012; Fernandes et al., 2013; Hokazono et al., 2013; Balk et al., 2014; Knier et al., 2016; Bsteh et al., 2020). Interestingly, both parameters can serve as biomarkers for disability progression, with suggested advantages for the latter (Bsteh et al., 2019a,b, 2021). In this context it needs to be noted that although there was a clear tendency toward a decrease in GCIPL and RNFLT in patients with MS-ON when compared to healthy control subjects, this effect did not reach level of significance. As previously mentioned, larger studies consistently report reduced GCIPL and RNFLT in patients with MS-ON, it is reasonable to suggest that the lack of statistical significance is related to the relatively small sample size of the current study (Oberwahrenbrock et al., 2012; Britze et al., 2017; Alonso et al., 2018; Balci et al., 2020; Farci et al., 2020).

Secondly, our results show that TRBF is lower in MS+ON eyes compared to MS-ON and healthy eyes. This clearly indicates that patients with a history of ON have compromised blood flow and supports the hypothesis that impaired blood flow and hypoperfusion may be an essential factor in patients with ON and MS (Kleerekooper et al., 2020). Again, our results are also in keeping with previous reports: Using a retinal function imager, Liu et al. showed that retinal perfusion is decreased in patients with relapsing MS when compared to healthy subjects (Liu et al., 2019). However, the latter study is limited by the fact that the authors did not differentiate between MS+ON and MS-ON eyes and the instrument used was not capable to provide data regarding TRBF. In another study, the same group of investigators assessed inter-eye correlations and potential differences of the retinal blood velocity in patients with MS (Jiang et al., 2016). The authors concluded that patients with MS show lower blood velocities as compared to healthy subjects (Jiang et al., 2016). Although the latter study measured only velocities but not volumetric blood flow as in the current study, this again supports our findings that blood flow is compromised in patients with MS. In addition, our finding of reduced blood flow is also compatible with the data of retinal vessel analysis as measured in the current study, showing a reduced AVR mainly caused by reduced CRAE. This, in turn, indicates pronounced arterial constriction in the major retinal vessels, which is in line with data on upregulation of endothelin-1 in MS lesions and elevated serum and CSF levels of this vasoconstrictive peptide (Speciale et al., 2000; Haufschild et al., 2001; Halder and Milner, 2021).

Finally, the finding of impaired volumetric blood flow in patients with MS is also compatible with the OCTA data of the current study. Our results show a pronounced decrease of capillary density in patients with MS, which again is more pronounced in MS+ON eyes compared to MS-ON eyes or healthy controls. This is also in keeping with previously published studies, which consistently reported a rarefication of the macular and/or peripapillary microvasculature in patients with MS (Feucht et al., 2019; Murphy et al., 2020; Yilmaz et al., 2020). Although OCTA does not allow for direct measurement of volumetric blood flow, it supports the hypothesis that microvascular perfusion may be impaired in patients with MS.

There is increasing evidence that mitochondrial dysfunction plays an important role in several neurodegenerative disorders, including MS (Mao and Reddy, 2010; Barcelos et al., 2019). Given the high metabolic rate of the neural tissue, an impairment of intracellular energy metabolism may easily translate to metabolic stress with the ultimate consequence of neurodegeneration (Mao and Reddy, 2010). Currently, there is no evidence regarding a direct interaction of MS-related mitochondrial dysfunction and decreased retinal oxygen extraction as observed in the current study. However, reduced oxygen metabolism in the retina might be an early sign of an impairment of neural function and may therefore serve as an additional retinal biomarker for disease progression complementing the purely structural parameters such as RNFLT and GCIPL.

Altered retinal oxygen metabolism has consistently been reported also for other neurodegenerative diseases than MS, such as mild cognitive impairment or Alzheimer’s disease (Olafsdottir et al., 2018; Stefánsson et al., 2019; Szegedi et al., 2020a), accompanied by a reduction of blood flow (Szegedi et al., 2020a) and microvascular dysfunction (Chua et al., 2020). This supports the hypothesis that oxygen extraction is related to neuronal degeneration or an impairment of neural function. Along this line of thought is has been shown that there is an age-dependent decline of retinal oxygen extraction correlated to the physiological age-dependent ganglion cell loss (Bata et al., 2019).

Our study has several strengths and limitations that warrant further discussion. The strength of our study is the use of a combination of state-of-the-art technology that allows us to draw direct conclusions regarding the oxygen metabolism of the retina. We have successfully used the same approach to assess oxygen extraction in healthy subjects (Palkovits et al., 2014; Werkmeister et al., 2015) as well as in patients with systemic diseases such as diabetes (Fondi et al., 2017), showing for the latter a reduction of oxygen extraction already in early disease stage.

Some limitations need to be addressed as well: First, our study is cross sectional in design. Therefore, based on the current results, it cannot be determined whether decreased blood flow and reduced oxygen extraction of the retinal neural tissue is a causative factor in the pathogenesis of the disease making the eye more vulnerable to damage or a consequence of retinal nerve fiber loss and a reduced oxygen demand of the tissue. Longitudinal studies would be necessary to finally get insight in this question and could also elucidate whether the assessed parameters are subject to temporal change and/or are associated with disease progression. Secondly, the study population is limited to a total number of 34 subjects. A larger study population would increase the power and allow for the detection of mores subtle changes in anatomical and functional properties of the retina especially in MS-ON patients. However, as the equipment used in the current study is not commercially available and requires particular training for the investigator, larger multicenter trials will be dependent on the future commercial availability of devices for measurement of ocular blood flow.

In summary, our data indicates that structural alterations found in the retinal tissue of patients with MS are accompanied by metabolic changes. Both oxygen metabolism and retinal blood flow seem to be impaired in patients with MS and history of ON. Whether this is a cause or a consequence of the disease has yet to be investigated.

## Data Availability Statement

The raw data supporting the conclusions of this article will be made available by the authors, without undue reservation.

## Ethics Statement

The studies involving human participants were reviewed and approved by Ethics Committee of the Medical University of Vienna. The patients/participants provided their written informed consent to participate in this study.

## Author Contributions

MK, DS, and GG contributed to conception and design of the study and drafted the manuscript. MK, NH, AS, GB, and PA were involved in acquisition of data. MK, GB, PA, AP-C, MP, RW, DS, LS, and GG performed the data analysis and interpreted the data. All authors contributed to manuscript revision and approved the submitted version.

## Conflict of Interest

The authors declare that the research was conducted in the absence of any commercial or financial relationships that could be construed as a potential conflict of interest.

## Publisher’s Note

All claims expressed in this article are solely those of the authors and do not necessarily represent those of their affiliated organizations, or those of the publisher, the editors and the reviewers. Any product that may be evaluated in this article, or claim that may be made by its manufacturer, is not guaranteed or endorsed by the publisher.
